# Management of hypersensivity pneumonitis

**DOI:** 10.1186/2045-7022-3-5

**Published:** 2013-02-04

**Authors:** Ioana O Agache, Liliana Rogozea

**Affiliations:** 1 Theramed Medical Center, Spatarul Luca Arbore 16, 500112, Brasov, Romania; 2 Faculty of Medicine, Transylvania University Brasov, Department of Allergy and Clinical Immunology, 500112, Brasov, Romania; 3 Faculty of Medicine, Transylvania University Brasov, Department of Fundamental and Prophylactic Medical Science, 500112, Brasov, Romania

**Keywords:** Hypersensitivity pneumonitis, Interstitial lung disease, Lung immune response, Drug-induced lung disease, Granulomatous inflammation

## Abstract

Hypersensitivity pneumonitis (HP) is an interstitial lung disease due to a combined type III and IV reaction with a granulomatous inflammation, caused by cytotoxic delayed hypersensitivity lymphocytes, in a Th1/Th17 milieu, chaperoned by a deficient suppressor function of T regulatory cells. Skewing toward a Th2 phenotype is reported for chronic HP. Phenotypic expression and severity depends on environmental and/or host genetic and immune co-factors. The wide spectrum of causative antigens is continuously up-dated with new sources of airborne organic particles and drug-induced HP. The diagnosis requires a detailed history, measurement of environmental exposure, pulmonary function tests, imaging, detection of serum specific antibodies, broncho-alveolar lavage, antigen-induced lymphocyte proliferation, environmental or laboratory-controlled inhalation challenge and lung biopsy. Complete antigen avoidance is the best therapeutic measure, although very difficult to achieve in some cases. Systemic steroids are of value for subacute and chronic forms of HP, but do not influence long term outcome. Manipulation of the immune response in HP holds future promise.

## Introduction

**Figure 1:**
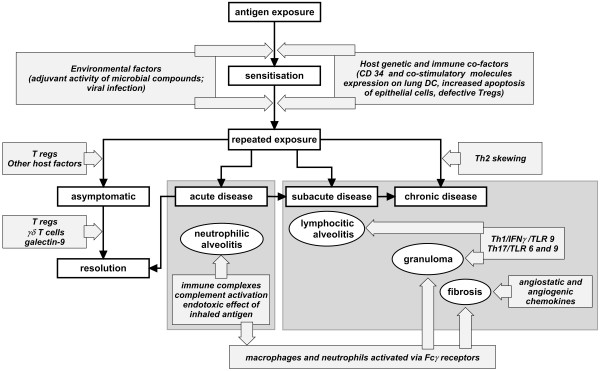
Pathogenenic mechanisms in hypersensivity pneumonitis.

Hypersensitivity pneumonitis (HP) is an interstitial lung disease (ILD) due to a combined type III and IV reaction with a granulomatous inflammation. The formation of immune complexes with IgG was considered to be a central part of pathogenesis. The current view is that HP is caused by cytotoxic delayed hypersensitivity lymphocytes, in a Th1/Th17 milieu (Figure 1).

Although many individuals are exposed to environmental antigens known to induce HP only approximately 5–15% will develop the disease [1]. The low prevalence rate suggests that the phenotypic expression of the disease depends on environmental co-factors, such as viruses [2,3] and/or host genetic and immune co-factors, which also promote considerable variability in disease severity and response to treatment. The mechanisms of progression to a chronic form remain unclear. For chronic HP a skewing toward a Th2 phenotype was described [4,5].

HP can occur in an occupational setting or after home exposure, following inhalation of organic antigens [mammalian and avian proteins, fungi, bacteria], low-molecular-weight chemicals or *Mycobacterium avium-intracellulare* complex organisms. As a non-inhalant variant HP can appear as a manifestation of drug-induced lung disease.

### Recently described entities

There is a wide spectrum of causative antigens for HP, and new sources of airborne organic particles are continually being recognized. Recently described are the trombone player and Chacinero’s lung [6,7], HP associated with catechin-rich green tea extracts [8], use of ultrasonic misting fountains at home [9], *Shiitake* mushroom spores [10], mosquito-coil smoke [11] medium-density fiberboard [12] or cash handling [13].

Reports on drug-induced HP are increasing in frequency, and interestingly some of these drugs were previously proposed as potential therapeutic agents for HP. Most of the recent reported drugs inducing HP are immune modulators used to treat neoplastic [14,15] and connective tissue diseases [16] or transplant recipients [17,18].

### Pathogenetic mechanisms relevant for future forms of therapy

a. The role of antigen-presenting cells

Through their key role in antigen presentation dendritic cells (DCs) are key players in the development of T cell–dependent adaptive immune responses. In an animal model of HP expression of stem cell antigen CD34 by lung mucosal DCs was required for migration of DCs from the lung to the lymph nodes in response to the HP antigen *Saccharopolyspora rectivirgula*. Loss of CD34 protected from development of HP. Since the CD34 molecule seems to play a role in the transition from a primary to a chronic T-cell response in HP and its deletion in mice did not associate any significant defect it might represent an appealing future therapeutic target in HP [19]. Another requirement for an efficient antigen presenting cell (APC) is the presence of adjuvant activity of the microbial components. IL-1 level is increased in the broncho-alveolar lavage fluid (BALF) of HP patients [20]. In mice models IL-1β demonstrated adjuvant properties [21], and was necessary for the production of antibodies against T-cell dependent antigens [22]. IL-1β acts directly on memory T Cells [21] and on CD4 T cells to enhance their antigen driven expansion and differentiation, especially into IL-17 and IL-4 producing cells [23]. Viral infection can also promote maturation of DCs [3].

HP is characterized by an influx of activated T cells to the lung, in which the CD28/B7 co-stimulatory signals from the APCs are essential for T cell activation and the outcome of the inflammatory response. The alveolar macrophages isolated from patients with HP have elevated levels of the co-stimulatory molecules CD80 and CD86 [24], while blockade of the CD80/86–CD28 co-stimulation pathway confers protection from experimental HP [25].

b. The role of T-cells

Interferon gamma [IFN-γ] plays a critical role in the formation of granuloma in HP, while TLR9 appears also required [26,27]. Overproduction of other Th1 cytokines, IL-12 and IL-18 by BALF macrophages was reported [28]. Other studies using animal models have suggested that HP is a Th17 disease calling into question the role of Th1 skewed response [29]. Genetic deletion and immunoneutralization of IL-17 protects against the development of mouse HP [30]. TLR9 is required for the development of Th17-mediated granulomatous inflammation [31]. Within the Th17 inflammatory milieu TLR6 plays a pivotal role in the development and severity of HP via its role in IL-17A production [32]. A recent study showed that initially IFN-γ production is dependent on IL-18 and the transcription factor T-bet, however as the disease continues IFN-γ production becomes IL-18-independent and partially T-bet dependent. In animal models for HP T-bet deficiency leads to a more severe disease characterized by an exacerbated Th17 cell response, decreased Th1 cell response, and increased collagen production in the lung. T-bet-mediated protection does not appear to be due to the development of a protective Th1 response because shifting the balance from a Th17 predominant response to a Th1 response by inhibition of IL-6 also results in lung pathology. Thus, both Th1 and Th17 cells can be pathogenic in HP, but they have divergent roles in the disease process [33].

In a mouse model of HP that progresses to lung fibrosis upon repeated exposure to *Bacillus subtilis*, γδ T cells expand in the lung and inhibit collagen deposition. In this model a subset of γδ cells are the predominant source of the Th17 cytokine IL-22. Blockade of expression of IL-22 accelerated lung fibrosis, whereas administration of recombinant IL-22 inhibited lung fibrosis. Moreover, the presence of protective γδ T cells and IL-22 diminished recruitment of CD4+ T cells to lung [34]. In the same model In the absence of IL-17 receptor signaling mice had delayed clearance of *Bacillus subtilis* with increased lung inflammation and fibrosis. Although IL-17A was predominantly expressed by γδ T cells, a compensatory increase in IL-17A expression by CD4[+] T cells was seen in the absence of γδ T cells that resulted in similar levels of IL-17A in the lungs in TCR δ deficient mice [35]. Galectin-9 was also proven to expand the immunosupressive macrophages and ameliorate experimental Th1/Th17 cell-mediated HP [36].

Loss of T-regulatory cells (Tregs) control over the immune response is essential for the impaired immune tolerance in HP. Experimental HP induced in CD4 + CD25+ Tregs-depleted mice showed a protective role of Tregs via suppression of IFN-γ production by T cells [37]. In humans T regs from BALF and blood obtained from asymptomatic exposed subjects had lower suppressive function compared to normal subjects, while Tregs from HP patients were totally nonfunctional and unable to suppress proliferation. Partially preserved Tregs suppressive function may explain antigen tolerance in asymptomatic exposed subjects. Defective Tregs function is potentially caused by increased IL-17 production since low levels of IL-17 were detected in sera and BALF from both normal and asymptomatic individuals, whereas measurable levels were found in HP patients [38].

c. The role of inflammation and apoptosis

Macrophages and neutrophils are activated in HP via Fc-γ receptors and accumulate in involved tissues [39]. Activated neutrophils loaded with matrix metalloproteinase 9 and collagenase-2 were found to play role in lung damage and fibrotic response in chronic HP [40]. In addition, angiostatic and angiogenic chemokines promote the development of fibrosis [41,42].

Increased apoptosis in non-hematopoietic cells and Gr-1+ granulocytes of the lungs promotes HP by enhancing maturation and chemokine production of CD11c + DC [43]. Immunohistochemical studies of surgical lung specimens from HP patients showed up-regulation on epithelial cells of Fas, Fas ligand, p53 and p21 expression in usual interstitial pneumonia (UIP)-like lesions compared with nonspecific interstitial pneumonia (NSIP)-like lesions. The expression of p53 and p21 was also increased in fibrotic NSIP [fNSIP]-like lesions compared with normal lung tissues [44].

### Diagnostic procedures

**Figure 2:**
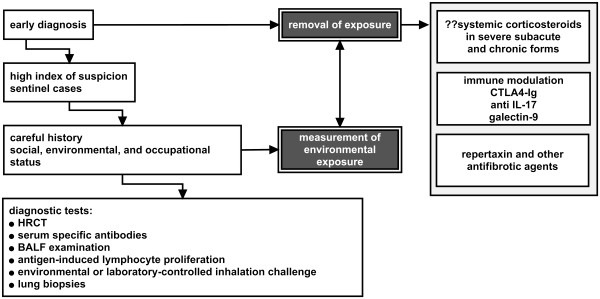
Management of hypersensivity pneumonitis.

At the current time, there is no single diagnostic single procedure or biomarker to confirm the diagnosis of HP. The diagnosis requires a detailed and careful history that would include social, environmental, and occupational status, measurement of environmental exposure, pulmonary function tests, imaging, detection of serum specific antibodies, examination of BALF, antigen-induced lymphocyte proliferation, environmental or laboratory-controlled inhalation challenge with the suspected antigen and lung biopsy (Figure 2). Major and minor diagnostic criteria are described [45]. A sentinel case should prompt to the identification of exposed subjects who might develop the disease. Improvement of symptoms away from exposure and/or a rapid response to oral steroids should heighten the awareness of HP.

a. Environmental exposure

Recognition of the exposure to antigen is critical for diagnosis and avoidance of exposure. The detection of a single causative antigen for HP can prove very difficult since usually subjects are exposed to several inhalation antigens.

For bird fancier’s lung (BFL) a variety of indirect and/or unrecognized exposures to birds were described including pigeons raised by neighbors, a flock of pigeons and/or wild birds in parks, shrines and railway stations [46,47]. In addition avian antigen was reported to persist in the patient’s house 6 months after removal of all birds and environmental cleanup [48].

Measuring the smallest amount of causative antigen in patient’s environment is crucial. For BFL the methods used to detect the avian antigen in dust samples (inhibition ELISA) [48], or in dust and air samples (direct competitive ELISA) [49], were not sensitive enough to measure a small amount of antigen. Kuramochi et al. recently described an antigen-capture ELISA with signal amplification using catalyzed reporter deposition for measuring small amounts of indoor and outdoor avian antigen [50]. Molecular methods using quantitative polymerase-chain reaction might offer advantages over culture and optical methods for estimating low level exposure to microbiological agents such as fungi [51-53].

Evaluation of personal exposure at work should include details on current and previous occupations, work processes, type of exposure and environmental factors. In farmer’s lung disease (FLD) both environmental factors and agricultural practices can independently modify concentrations in hay of microorganisms potentially responsible for HP. Bad climatic conditions of harvest, high-density hay-packing modes, (especially round balls) and altitude (700–900 m) were associated with high concentrations of microorganisms in hay [54]. Worksite assessment together with an industrial hygienist is recommended.

b. Imaging

High resolution computed tomography (HRCT) is the “gold-standard” imaging method for HP. Although the individual HRCT findings are nonspecific, their combination coupled with their distribution can narrow the differential diagnosis. There are corresponding findings for each of the clinical phases of HP, although there is some overlap between the phases. Typical findings include confluent opacities in the acute phase, centrilobular nodules, areas of ground-glass attenuation, a mosaic perfusion pattern, air trapping in the subacute phase and subpleural irregular linear opacities with associated architectural distortion in the chronic phase. The reticulation HP can be random or have a predominantly subpleural and peri-bronchovascular distribution. Honeycombing may be present. In the acute and subacute phases, the disease is predominantly in the lower lungs, whereas in chronic HP the findings are predominant in the mid to upper lungs [55].

HRCT can be normal, especially when scans are obtained at greater intervals in relation to exposure [56].

c. Serum specific antibodies

The finding of circulating antibodies against the putative offending antigens is useful for diagnosis. However, it is important to emphasize that exposed individuals may have antibodies without disease, and some patients may give false negative results [57], diagnostic panels are usually incomplete and lab techniques may be insensitive to detect low levels of antibodies. Enzyme immunoassays (EIA) have successfully replaced in many cases the precipitation techniques. However, standardising EIA proves challenging because of the difficulty in obtaining stable solid phases from a huge variety of heterogeneous antigenic sources. Novel detection methods such as the automated fluorimetric EIA (Streptavidin ImmunoCAP) have been advocated for measuring antibodies elicited by protein antigens that are not commercially available [58]. The method can also differentiate with a specificity of 97% and a sensitivity of 90% between IgG antibodies specific for feather duvet lung (FDL) and IgG specific for BFL [59]. The use of recombinant antigens was reported for serological diagnosis of metalworking fluid-associated HP (MWF-HP) [60].

Choosing the relevant antigens for the EIA detection panel is essential. Out of the three *Eurotium amstelodami* antigens extracts tested (ascospore, conidia and hyphae), the cutoffs showed the highest sensitivity and specificity for the ascospore antigen, thus its measurement improves significantly the serological diagnosis of FLD [61]. Instead of using a standard kit the selection of antigens to be tested is better determined locally according to the prevalent antigens [62].

The sensitivity and specificity of serologic tests varies with the clinical form of the disease. For example, in acute BFL antibodies titers were markedly increased and showed high sensitivity and specificity ranging from 75–100%, while in the chronic form antibody reactivity was slightly increased, showing a sensitivity of 27–73% and specificity of 45–100% [63].

The discriminative value of serologic tests between exposure and disease is controversial, and might vary with the putative antigen and with the detection method. One study showed no difference between HP patients and exposed controls for serum concentrations of IgG against moulds or mammals antigens [64]. However, in the same study IgG against bird antigens were significantly higher in HP patients versus exposed controls. In contrast, an older study using an immunoprecipitation method reported an incidence of 40% for specific IgG in asymptomatic bird keepers [65]. A threshold was proposed to discriminate between exposed controls and MWF-HP, but this remains to be proven for other antigens [66]. For antibodies against molds electrosyneresis on cellulose acetate was suggested to discriminate best between active HP and other types of ILD [67], or between patients and healthy exposed farmers [68].

Whether antibodies measured in healthy exposed controls further predispose to full blown HP remains to be proven in longitudinal studies. It has been shown that patients who were exposed to avian inhalation antigens in their childhood or during later life may develop FDL after exposure to bedding filled with goose feathers [69].

d. Bronchoalveolar lavage fluid examination

A normal BALF excludes HP diagnosis [70]. On the other hand asymptomatic exposed subjects can have a lymphocytic alveolitis, which is not part of a subclinical form of disease as proved by the long-term follow up of these individuals [65].

BALF examination in HP usually shows a lymphocytic alveolitis, classically with an increase of CD8+ lymphocytes and inversion of the CD4/CD8 ratio, associated with moderate neutrophilia, and mild eosinophilia and mastocytosis [70]. An increase in plasma cells, signs of macrophage and T cell activation, foamy macrophages or with cockade-like structures in their cytoplasm were also described [71-74]. Acute episodes of HP are associated with an influx of neutrophils lasting for up to 1 week. After this period, the cellular profile of the BALF returns to lymphocytic alveolitis.

Absence of a low CD4/CD8 ratio should not exclude HP diagnosis, since a recent study reported a low ratio in only 34% cases. The CD4/CD8 ratio was not different between forms, etiologies of HP, and time elapsed since last antigen exposure, but was higher in women [75].

Immunophenotyping of BALF cells might improve diagnostic accuracy and measure disease activity. Thus, for HP patients a higher proportion of CD8 + T-cytotoxic cells was described. For all ILD a positive association was found between the density of type I alveolar epithelial cells and FVC [76]. Increased expression of CD69, VLA-1 and decreased expression of CD28 on CD4+ cells and increased expression of HLA-DR on CD8+ cells was correlated with the extent of parenchymal involvement and decreased DLCO values [77].

The detection of fungal DNA in BALF cell pellets might prove an useful diagnostic tool [78]. Proteome analysis of BALF in chronic HP pinpointed several biomarkers which might distinguish between the UIP and fNSIP patterns [79].

e. Antigen-induced lymphocyte proliferation

Antigen-induced lymphocyte proliferation can aid the diagnosis of insidious forms of HP. In FDL specific antibodies against avian antigens were positive only in the acute form of the disease, while antigen-induced lymphocyte proliferation in peripheral blood or BALF cells was positive in all the patients [80]. In chronic BFL specific antibodies against pigeon or budgerigar dropping extracts were positive in 87% of the recurrent form and 35% of the insidious cases, while antigen-induced lymphocyte proliferation was positive in more than 90% of both groups [46]. Antigen-induced lymphocyte proliferation is also useful in differentiating subjects with HP from asymptomatic exposure [81,82].

f. Environmental or laboratory-controlled inhalation challenge

The use of inhalation challenge tests for HP diagnosis is hampered by the lack of standardization for the inhalation protocol and for defining a positive response.

The reported diagnostic sensitivity of the test for BFL is 92%, while specificity reaches 100% [83]. Inhalation challenge with avian dropping extracts in chronic BFL evaluated as positive or probable all patients further proven by a combination of diagnostic criteria, whereas all control subjects were evaluated as negative [84].

g. Lung biopsies

Establishing the diagnosis of HP in the absence of lung biopsy is challenging and is heavily dependent on identification of a specific antigenic exposure. Examination of surgical lung biopsies in HP usually shows an airway-centered chronic interstitial pneumonia, a lymphocyte-rich chronic bronchiolitis, and poorly formed non-necrotizing granulomas distributed mainly within the peribronchiolar interstitium. Variable degrees of peribronchiolar fibrosis and hyperplasia of the bronchiolar epithelium are described. In some patients, granulomatous inflammation may be lacking, resulting in a histological appearance resembling NSIP [85]. Increased fibrin deposition and neutrophilic infiltrate suggests either acute HP or exacerbation of chronic HP [86]. Late-stage fibrotic HP closely mimics UIP or fNSIP, while cellular NSIP or organizing pneumonia patterns are rarely found [85,87]. Centrilobular fibrosis, often connecting to the perilobular areas in the appearance of “bridging fibrosis” in association with an UIP pattern is also suggestive for chronic HP [88]. Acute exacerbations of HP appear as diffuse alveolar damage or bronchiolitis obliterans organizing pneumonia superimposed upon the fibrosing interstitial pneumonia [89].

Immunohistochemical demonstration of the causative antigen in the lung tissue might prove valuable. For BFL immunohistochemistry using a polyclonal antibody against pigeon serum showed a predominant cytoplasmic immunostaining in multinucleated giant cells and histiocytes from lung granulomas [90]. Immunohistochemistry can identify microgranulomas in chronic HP following detection of cathepsin-K, a potent cysteine protease expressed at high levels in activated macrophages [91].

### Measuring HP activity

An increase in alveolar NO following re-exposure in HP was described in a case-report [92] and deserves further investigation as a measure of disease activity in HP.

As biomarkers reflecting the lung injury/regeneration cycle substances derived from type II pneumocytes are potential candidates to monitor disease activity. Serum Krebs von den Lungen-6 mucin (KL-6) is elevated in 70–100% of patients with various ILDs, including HP [93]. Increase in serum level of surfactant protein D [SP-D] was also reported [94]. In a long term follow-up of a patient with BFL after removal of antigen exposure SP-D and KL-6 returned to normal in 8 and 18 months respectively, while DLCO improved slowly in parallel [95].

### Prognostic factors

The long-term outcome (mean 14 years) of pulmonary function was evaluated in FLD patients compared to control farmers matched by age, sex, and smoking habits. The mean DLCO was on average with 12% lower in patients compared to controls. The mean maximum expiratory flow at 50% of vital capacity was also lower and airway obstruction was more common. Patients with recurrent episodes had a significantly lower mean DLCO compared to those experiencing only a single episode [96].

High BALF neutrophil count was documented to be a sign of irreversible lung fibrosis [32]. CA 15–3 was demonstrated equally sensitive and specific to KL-6 in terms of differentiating between ILDs with and without fibrosis [97].

As for other ILD the presence of established fibrosis, especially when associated with architectural distortion in the form of honeycombing, is associated with shorter survivals [98]. HRCT patterns, in particular, severity of traction bronchiectasis and extent of honeycombing are superior to FEV1, FVC and DLCO for predicting mortality in patients with chronic HP [99]. The prognosis of fNSIP pattern tends to be better compared to the UIP pattern [87]. Pulmonary hypertension is not rare in chronic HP and significantly impacts survival [100].

Patients with low total lung capacity and DLCO, low lymphocyte levels in BALF and a UIP-like pattern at the time of diagnosis have increased risk for acute exacerbations of chronic HP [101].

The 10.6% reported prevalence of lung cancer in chronic HP is similar to idiopathic pulmonary fibrosis (IPF). Tumors were located adjacent to honeycombing lesions, bullae or in the relatively normal lung [102].

### Treatment

Complete antigen avoidance is the essential step in the management of HP (Figure 2). The majority of cases improve or heal, but some evolve to a chronic form probably due to persistence of exposure at an undetectable level in association with genetic and immunologic factors. In a long term follow-up of patients with BFL there was persistence of sensitized lymphocytes and antibody production in the respiratory tract up to 5 years [103].

Systemic corticosteroids are recommended for subacute and chronic forms of HP. The usual regimen consists of initial high doses followed by gradual tapering. Treatment continues until no further improvement in physiologic abnormalities is observed. The use of inhaled corticosteroids is anecdotal.

In a cohort of Danish children with HP high dose intravenous methylprednisolone had no clinical impact, since both lung function and DLCO remained subnormal [104]. In adults with FLD corticosteroid treatment improved quickly lung function compared to placebo, but after 5 years there was no difference between the two groups. An increased incidence of recurrent attacks, without reaching statistical significance, was observed in the corticosteroid group [105]. In adults with chronic HP prednisolone was effective in only 58% of cases [106], thus for progressive chronic HP immunosuppressants may be necessary.

Although in vitro studies have shown promise for thalidomide [107], pentoxifylline [108], low-dose long-term macrolides [109], and cyclosporine [110], no controlled clinical trials have been performed. In addition, several recent reports on HP induced by thalidomide-like drugs such as lenalidomide [111,112], roxytromycin [113], cyclosporin [114] and cyclosporin-like drugs such as everolimus [17] and sirolimus [18] preclude the use of these drugs for HP. Given the role of IFN-γ in promoting granuloma formation in HP it cannot be an option as antifibrotic treatment as it is for IPF.

Manipulation of the immune response in HP holds future promise. In a HP experimental model mice treated with CTLA-4Ig showed a significant decrease in the extent of lung damage and in the number of BALF inflammatory cells, with diminished CD4/CD8 T cell ratio. A significant increase in the lung γδT and NKT cells was observed after two weeks of CTLA-4Ig administration, while after 3 weeks an increased of regulatory T cells occurred [115]. Other potential targets for immune modulation in HP are Tbet, DCs and adjuvant factors, Th17, galectin-9, Tregs and activated cytotoxic T cells.

Reparixin (Repertaxin)™ (Dompe Farmaceutici S.p.A., Milan, Italy Product), an inhibitor of CXCR1 and CXCR2 activation, has been shown to attenuate inflammatory responses in various injury models [116,117] and might prove useful in managing pulmonary fibrosis.

### Key-messages

1. new antigens inducing HP are described every year for both home and work exposure

2. high index of suspicion for HP in cases with unexplained dyspnea and frequent monitoring of subjects at risk are essential for an early diagnosis

3. a set of complementary diagnostic tests should be used for diagnosis and to assess severity, disease activity and prognosis

4. environmental detection of antigen is essential for both diagnosis and further avoidance of exposure

5. future improvement of the serological diagnosis of HP needs a better inventory of etiologic agents with generation of local relevant diagnostic panels, the use of more efficient serological techniques, together with longitudinal evaluation of exposed subjects.

6. complete antigen avoidance is the best therapeutic measure, although very difficult to achieve in some cases

7. systemic steroids are of value for subacute and chronic forms of HP but do not influence long term outcome

8. novel anti-inflammatory, immunoregulatory, and antifibrotic treatments are urgently needed for subacute and chronic HP

## Abbreviations

APC: Antigen presenting cells;BALF: Broncho-alveolar lavage fluid;BFL: Bird-fancier lung;CTLA-4: Cytotoxic T-Lymphocyte Antigen 4;DCs: Dendritic cells;DLCO: Diffusing capacity;EIA: Enzyme immunoassays;FDL: Feather duvet lung;FEV1: Forced expiratory volume in the first second;FLD: Farmers’ lung disease;fNSIP: Fibrotic nonspecific interstitial pneumonia;FVC: Forced vital capacity;HP: Hypersensitivity pneumonitis;HRCT: High resolution computed tomography;IFN-γ: Interferon gamma;Ig: Immunoglobulin;ILD: Interstitial lung disease;IPF: Idiopathic pulmonary fibrosis;KL-6: Serum Krebs von den Lungen-6 mucin;MWF-HP: Metalworking fluid-associated HP;NKT cells: Natural killer T cells;NO: Nitric oxide;NSIP: Nonspecific interstitial pneumonia;SP-D: Surfactant protein D;UIP: Usual interstitial pneumonia;Tregs: T regulatory cells

## Competing interests

The authors declare no competing interests relevant to this paper.

## Authors’ contribution

IA, LR both equally contributed to the manuscript. Both authors read and approved the final manuscript.
